# Dynamic large-scale network synchronization from perception to action

**DOI:** 10.1162/netn_a_00039

**Published:** 2018-10-01

**Authors:** Jonni Hirvonen, Simo Monto, Sheng H. Wang, J. Matias Palva, Satu Palva

**Affiliations:** Helsinki Institute for Life Sciences, Neuroscience Center, University of Helsinki, Finland; BioMag Laboratory, HUS Medical Imaging Center, Helsinki University Central Hospital, Finland; Helsinki Institute for Life Sciences, Neuroscience Center, University of Helsinki, Finland; Helsinki Institute for Life Sciences, Neuroscience Center, University of Helsinki, Finland; Helsinki Institute for Life Sciences, Neuroscience Center, University of Helsinki, Finland; Helsinki Institute for Life Sciences, Neuroscience Center, University of Helsinki, Finland

**Keywords:** MEG, Synchronization, Somatosensory, Communication, Perception, Action

## Abstract

Sensory-guided actions entail the processing of sensory information, generation of perceptual decisions, and the generation of appropriate actions. Neuronal activity underlying these processes is distributed into sensory, fronto-parietal, and motor brain areas, respectively. How the neuronal processing is coordinated across these brain areas to support functions from perception to action remains unknown. We investigated whether phase synchronization in large-scale networks coordinate these processes. We recorded human cortical activity with magnetoencephalography (MEG) during a task in which weak somatosensory stimuli remained unperceived or were perceived. We then assessed dynamic evolution of phase synchronization in large-scale networks from source-reconstructed MEG data by using advanced analysis approaches combined with graph theory. Here we show that perceiving and reporting of weak somatosensory stimuli is correlated with sustained strengthening of large-scale synchrony concurrently in delta/theta (3–7 Hz) and gamma (40–60 Hz) frequency bands. In a data-driven network localization, we found this synchronization to dynamically connect the task-relevant, that is, the fronto-parietal, sensory, and motor systems. The strength and temporal pattern of interareal synchronization were also correlated with the response times. These data thus show that key brain areas underlying perception, decision-making, and actions are transiently connected by large-scale dynamic phase synchronization in the delta/theta and gamma bands.

## Author Summary

We studied the functional role of interareal phase synchronization in the perception-to-action cascade. To this end we recorded human cortical activity with magnetoencephalography (MEG) during a task where weak somatosensory stimuli were perceived or remained unperceived. We identified time-resolved large-scale networks of synchronization by using source-reconstructed MEG data with advanced analysis tools and graph theory. We found that perceiving and reporting of weak somatosensory stimuli was correlated with sustained strengthening of large-scale phase synchronization concurrently in delta/theta (3–7 Hz) and gamma (40–60 Hz) frequency bands. Synchronization characterizing the perceived stimuli connected the sensorimotor system with frontoparietal attentional systems. These data suggest that large-scale network synchronization may coordinate neuronal processing across brain regions during perception, decision-making, and when responding to weak somatosensory stimuli.

## INTRODUCTION

Understanding the neuronal mechanisms that govern the translation of conscious perception of sensory information into perceptual decisions and generation of appropriate actions is an important challenge in neuroscience. Several studies using functional magnetic resonance imaging (fMRI; Blankenburg et al., 2003; Dehaene and Changeux, 2011; Hegner et al., 2016; Li Hegner et al., 2015), human intracranial electroencephalography (iEEG; Fisch et al., 2009; Gaillard et al., 2009), and noninvasive source-reconstructed electro- and magnetoencephalography (EEG and MEG; Hirvonen & Palva, 2016; King et al., 2016; Salti et al., 2015) show that neuronal activity in prefrontal (PFC) and posterior parietal cortex (PPC) together with that in sensory areas is correlated positively with conscious perception. King et al. (2016) recently used a decoding approach of MEG data to show that brain regions as well as temporal evolution of neuronal processing were distinct for perception, maintenance, and visibility of seen and unseen visual stimuli.

Also, perceptual decision-making, for deciding to report the presence or absence of a sensory stimuli or a target, involves activity in PPC and PFC in human (Donner et al., 2009b; Kaplan et al., 2017; Tosoni et al., 2008), monkey (Siegel et al., 2015), and mouse (Goard et al., 2016) local field potential data. More specifically, it has been suggested that the sensorimotor decision process engages neuronal structures that are involved in preparing the associated actions, such as the oculomotor regions of parietal and prefrontal cortex in monkeys (Schall, 2001; Yang & Shadlen, 2007) and the response-related PPC regions and motor cortex in humans (Donner et al., 2009b; Gould et al., 2012; Kaplan et al., 2017; Tosoni et al., 2008) (Medendorp et al., 2011). Motor actions, conversely, dynamically modulate the processing of sensory information (Gutteling et al., 2011; Wohlschlager, 2000), and at the behavioral level, rhythmicity of visual perception is coupled with that of action (Tomassini et al., 2015).

These lines of evidence thus indicate multiple levels of coupling between perception and action, but how this cooperation is achieved at the neuronal level has remained incompletely understood. One candidate mechanism to achieve such coordination is neuronal synchronization, which has been shown to regulate neuronal communication (Fries, 2015; Gregoriou et al., 2009; Maris et al., 2016; Siegel et al., 2012; Singer, 2009) and provide a temporal correlation–based framework for integrating information processing in neuronal circuits at multiple scales (Fries, 2015; Schroeder & Lakatos, 2009; Siegel et al., 2012). In humans, neuronal activity in a large fraction of the cerebral cortex can be noninvasively recorded with EEG and MEG, but using these methods for estimating long-range synchronization demands both a source reconstruction–based approach and methods to address the challenges of residual signal leakage (Brookes et al., 2012; Palva & Palva, 2012; Schoffelen & Gross, 2009). Preliminary evidence from the visual modality suggests that long-range synchronization among the visual and frontal brain areas is indeed correlated with visual perception (Hipp et al., 2011) and that between motor cortical areas and peripheral nervous system with the execution of motor actions (Schoffelen & Gross, 2009). Although these studies provide partial support for the idea that perception-action cycle could be implemented via phase synchronization, the complete network architectures bridging perception and action have remained unaddressed.

**Figure 1.**
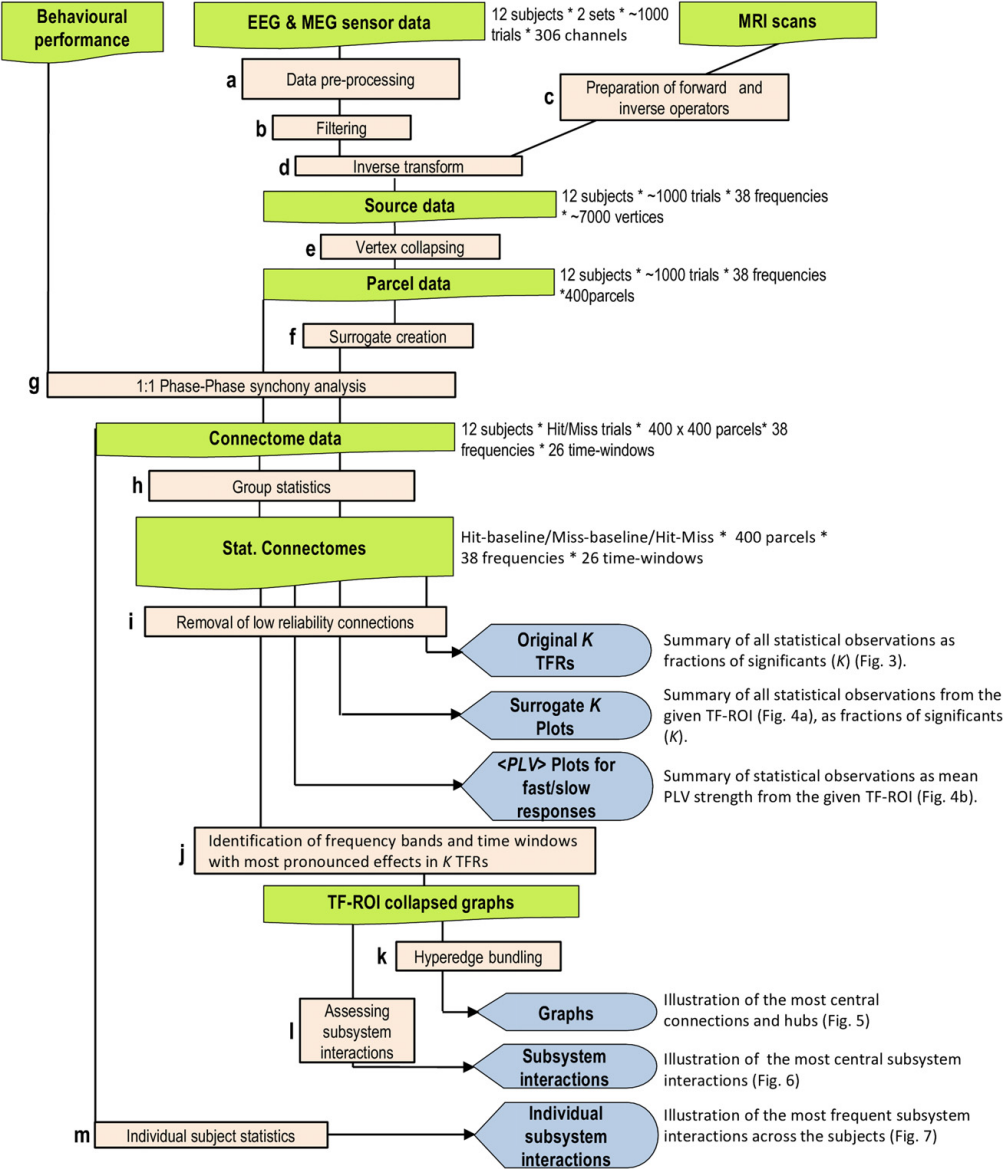
A schematic overview of the analysis pipeline. This figure shows the different analysis steps and outputs of the results (for a–m, see Methods).

We hypothesized that dynamic large-scale network phase synchronization would underlie the coordination of neuronal processing from perception to perceptual decisions and actions by regulating the neuronal processing between PFC, PPC, and sensory and motor systems. To test this hypothesis, we recorded MEG during a somatosensory stimulus detection task in which the subjects were presented constant-current somatosensory stimuli at the threshold of perception that were perceived or remained unperceived (Hirvonen & Palva, 2016). We then used MEG source modeling–based all-to-all phase coupling analysis (Figure 1) to identify the cortical networks characterizing perception-action sequence–associated neuronal processing. We found concurrent delta/theta- and gamma-band synchronization to connect the somatosensory, fronto-parietal, and motor cortical regions for stimuli that were perceived and an absence of this coupling for stimuli that remained undetected. These findings provide evidence for neuronal phase synchronization to play a functionally significant role in the cooperation of sensory, decision, and motor stages from perception to action (Figure 1).

## RESULTS

### Behavioral Performance

**Figure 2.**
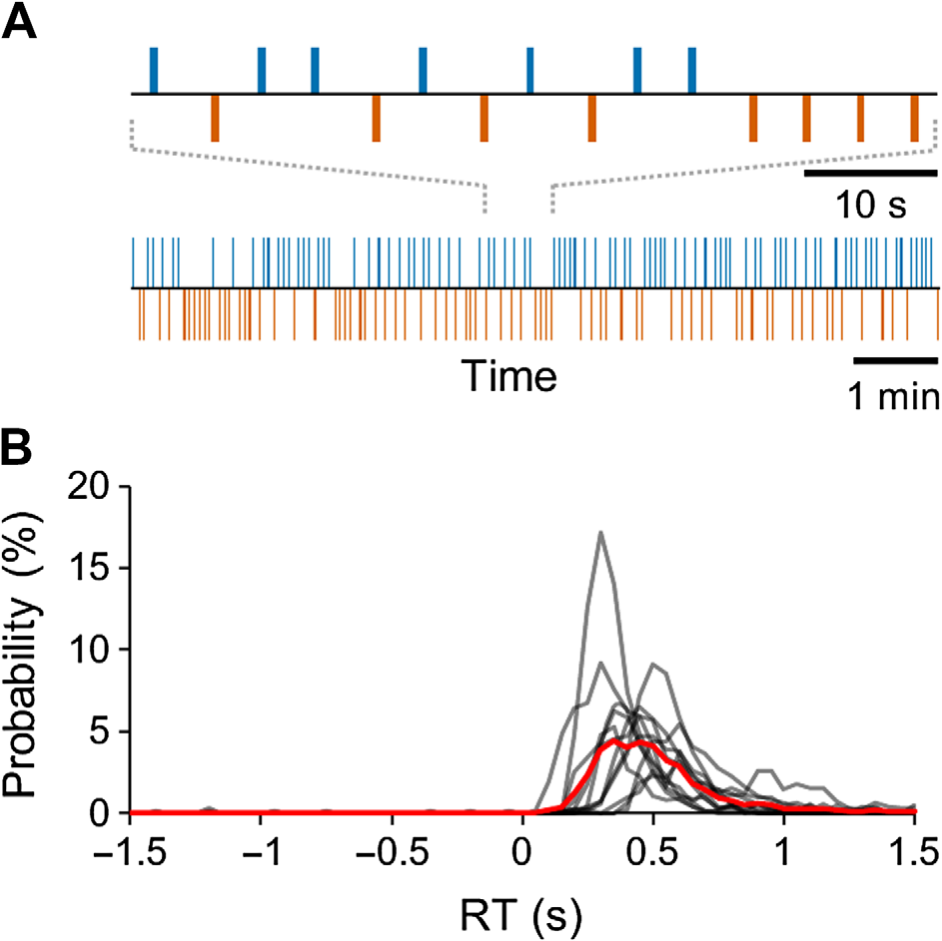
Task and behavioral performance. (A) Schematic illustration of the experimental para digm with a stream of constant-intensity somatosensory stimuli presented at 1.5- to 4.5-s intervals and the corresponding behavioral Hit-Miss time series for a representative subject. Blue bars denote consciously perceived stimuli, and the red bars denote unperceived stimuli. (B) Individual (black lines) and group (red line) response time (RT) distributions.

The subjects’ task during the MEG recordings was to detect weak, constant-intensity electrical stimuli given to the tip of the right index finger during two 30-min sessions at random intervals between 1.5 and 4.5 s. The strength of the stimuli was calibrated to the threshold of detection prior to recordings and maintained constant during the MEG recordings. Thus, during MEG recordings, these stimuli were variably either detected (Hits) or remained undetected (Misses) (Figure 2A). The hit rate (HR) was 38.32 ± 15.26% [mean ± standard deviation (SD)], and the reaction time (RT) was 399 ± 135 ms (mean ± SD). We estimated the fraction of false alarms (FA), that is, responses when no stimulus was presented, from the time window preceding electrical stimuli (Figure 2B, see Methods section). On the basis of the finite-state model approach, we estimated FA rate to be 0.2 ± 0.3% (mean ± SD) of all trials, which gives a true stimulus detection rate of 38.12%.

### Sustained Large-Scale Delta and Gamma Synchrony Characterizes Perception-Action Cycle

**Figure 3.**
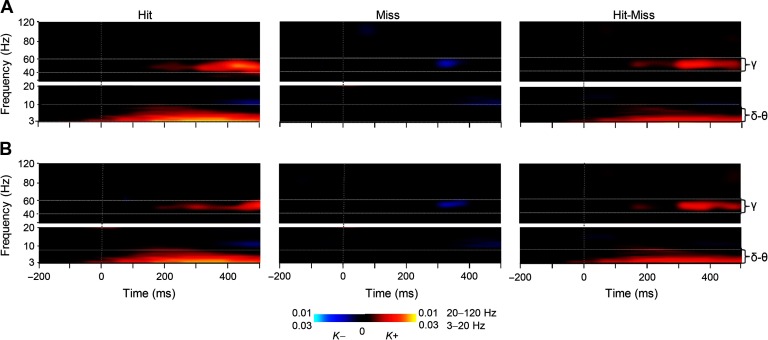
Large-scale delta- and gamma-band synchronization characterizes neuronal processing of perceived but not unperceived stimuli. (A) Time-frequency representations of the extent of significant interareal synchronization as estimated with PLV for perceived (Hit) stimuli, unperceived (Miss), as well as for their difference (Hit-Miss) compared with the prestimulus baseline (*N* = 12) (Wilcoxon signed-ranked test, *p* < 0.05, corrected for multiple comparisons). Sustained gamma-band (40–60 Hz) synchronization and delta/theta- (δ/θ, 3–7 Hz) band synchronization were stronger for Hits than Misses. The color indicates the connection densities of positive (*K*+) and negative (*K*−) observations, that is, the fractions of connections with a statistically significant positive or negative difference from the baseline level, respectively. (B) Time-frequency representations of the extent of significant interareal synchronization as estimated with iPLV.

To investigate whether large-scale synchronization would characterize the neuronal processing from perception to action and to map the anatomy and frequency patterns of such putative synchronization, we tested whether synchronization would characterize the processing of those stimuli that were reported perceived. We thus characterized phase synchrony among all cortical areas for each subject, time window, and frequency band separately for trials in which near-sensory stimulation was considered as perceived (Hits) or as unperceived (Misses). We represented statistically significant observations of interareal phase synchrony as graphs where cortical areas were the vertices and significant connections the edges (Bullmore & Sporns, 2009; Palva et al., 2010; Rubinov & Sporns, 2010). The extent of synchrony was estimated with connection density (*K*), which is the proportion of significant interareal interactions from all possible pairwise interactions among the 400 brain areas in our cortical parcellation. The frequency spectrum of *K* showed that synchronization in the mid-gamma frequency band (mid-*γ*, 40–60 Hz) at 150–500 ms and in the delta/theta (δ–θ, 3–7 Hz) band from 0 to 500 ms after the stimulus onset was strengthened above baseline levels for Hits (Wilcoxon signed-rank test, *p* < 0.05, corrected for multiple comparisons) when interareal synchronization was measured with the phase-locking value (PLV; Figure 3A). An essentially identical result was obtained when the imaginary part of PLV (iPLV) that is insensitive to source mixing was used to measure synchronization (Figure 3B), which showed that delta and gamma-band synchronization were not attributable to signal mixing and artificial synchronization. In contrast, interareal synchronization was largely insignificant (Wilcoxon signed-rank test, *p* < 0.05, corrected for multiple comparisons) for Misses and in the gamma band even briefly suppressed below the baseline level both when estimated with PLV (Figure 3A) and iPLV (Figure 3B). Importantly, this suppression shows that the lack of synchronization for Misses was not due to poor signal-to-noise ratio for missed stimuli, but that Hits and Misses were associated with genuinely distinct synchronization patterns. A comparison between Hits and Misses confirmed that both large-scale delta- and gamma-band synchrony were indeed stronger for Hits than Misses (Wilcoxon signed-rank test, *p* < 0.05, corrected) estimated with both PLV (Figure 3A) and iPLV (Figure 3B).

Observations of large-scale synchronization can also arise artificially from evoked responses or phase locking of ongoing activity to the stimulus onsets (Palva & Palva, 2012). In the present data, early transient phase locking of ongoing oscillations to stimulus onset as well as transient amplitude increase was observed for both Hits and Misses and was stronger for Hits than Misses between 3 and 30 Hz (Hirvonen & Palva, 2016) (Supporting Information Figure 2, Hirvonen, Monto, Wang, Palva, & Palva, 2018). Sustained phase locking of oscillations to stimulus onset and sustained oscillation amplitude increases during the time windows of large-scale delta/theta- and gamma-band phase synchronization were observed only in the low-alpha and delta/theta frequencies. In the delta/theta band both phase locking and oscillation amplitudes were increased, whereas in the alpha band, phase locking to stimulus onset was increased but the oscillations amplitudes were more suppressed for the Hits than for Misses.

**Figure 4.**
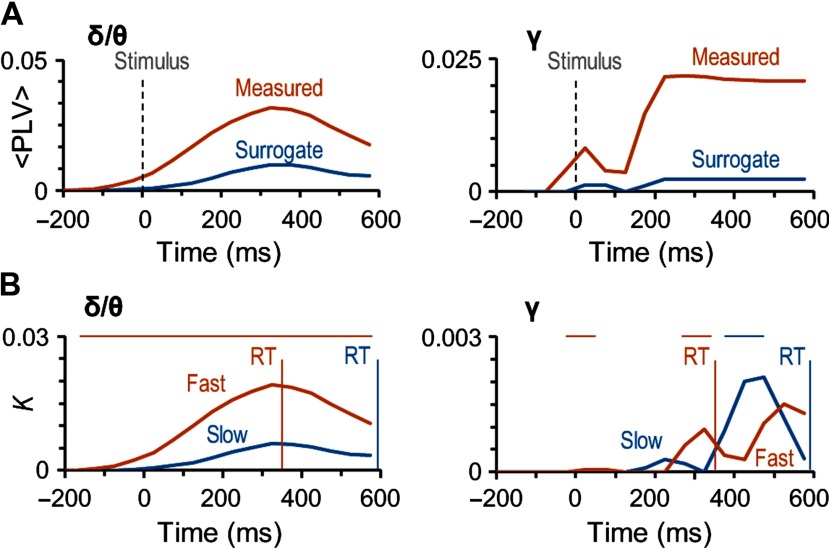
Gamma- and delta/theta-band synchronization is not artificial and predicts the speed of sensorimotor decisions. (A) The mean coupling strength (<PLV>) for the significant edges for Hits in the original (red line) and surrogate data (blue line) in delta/theta- (δ/θ) and gamma- (*γ*) frequency bands. (B) Connection density (*K*) as a function of time for the difference between Hits and Misses separately for trials with fast (red) and slow (blue) RTs. Vertical lines show the mean RT in these two categories: 356 ± 122 ms (mean ± SD across subjects) for the trials with fast RTs and 594 ± 229 ms for the trials with slow RTs. The horizontal bars above the plots show the time window in significant difference between trials with fast and slow trial RTs (Wilcoxon signed-ranked test, *p* < 0.05, corrected for multiple comparisons). The trials were split at the median RT of each subject and the mean of these median RTs was 464 ± 142 ms (mean ± SD across subjects).

These findings hence indicate qualitatively that large-scale gamma-band synchronization cannot be caused by evoked or stimulus phase-locked activity. To confirm this as well as to assess the contribution of evoked activity in the observed delta/theta band large-scale synchronization, we compared synchronization data against surrogate data where non-stimulus-locked phase relationships were eliminated but evoked/stimulus-locked components were preserved (see Methods). We compared the strengths of phase synchrony in original data against the corresponding mean values of surrogate data.Importantly, in the delta/theta (Figure 4A, left) and specifically in the gamma-band (Figure 4A, right) synchronization was significantly stronger in the original than surrogate data for Hits. These analyses thus unequivocally indicate that neither the large-scale delta/theta- or gamma-band synchronization observed for detected stimuli can be explained by artificial synchronization caused either by phase locking of ongoing oscillations to stimulus onset or additive evoked responses, or by confounders such as stimulus-altered local source topographies or autocorrelations.

### Correlation of Large-Scale Synchronization with the Motor Responses

Our main goal was to reveal the networks underlying the coordination of processing from perception to action. To thus identify whether and when synchronization is locked to actions, that is, the motor responses rather than related to perceptual processing, we investigated whether the strength of the observed large-scale delta/theta and gamma-band synchronization would be correlated with RTs. We divided the responses within each subject into “fast” (356 ± 122 ms) and “slow” (594 ± 229 ms) response categories by the individual median RT (464 ± 142 ms, mean ± SD across subjects). We then estimated the strength of synchronization separately for these two categories of responses. This analysis indicated that for fast and slow responses both delta/theta band and gamma bands had distinct synchronization patterns (Figure 4B). For in delta/theta, synchronization was stronger for the fast than for the slow responses during the whole response time (Wilcoxon signed-ranked test, *p* < 0.05, corrected for multiple comparisons). However, gamma-band synchronization differed for the fast and slow responses only during the different peak latencies, which showed that for the fast responses, gamma-band synchronization began around 200 ms from stimulus onset, whereas for the slow responses it began 350 ms from stimulus onset (Wilcoxon signed-ranked test, *p* < 0.05, corrected for multiple comparisons). These very different patterns of correlations with the RTs suggest that although delta/theta synchronization coordinates directly the motor actions, the functional significance of gamma-band synchronization in coordinating motor actions is dependent on perceptual and/or decision processes that have large temporal variability.

### Large-Scale Synchrony Connects Sensorimotor Areas with Prefrontal and Posterior Parietal Cortices

**Figure 5.**
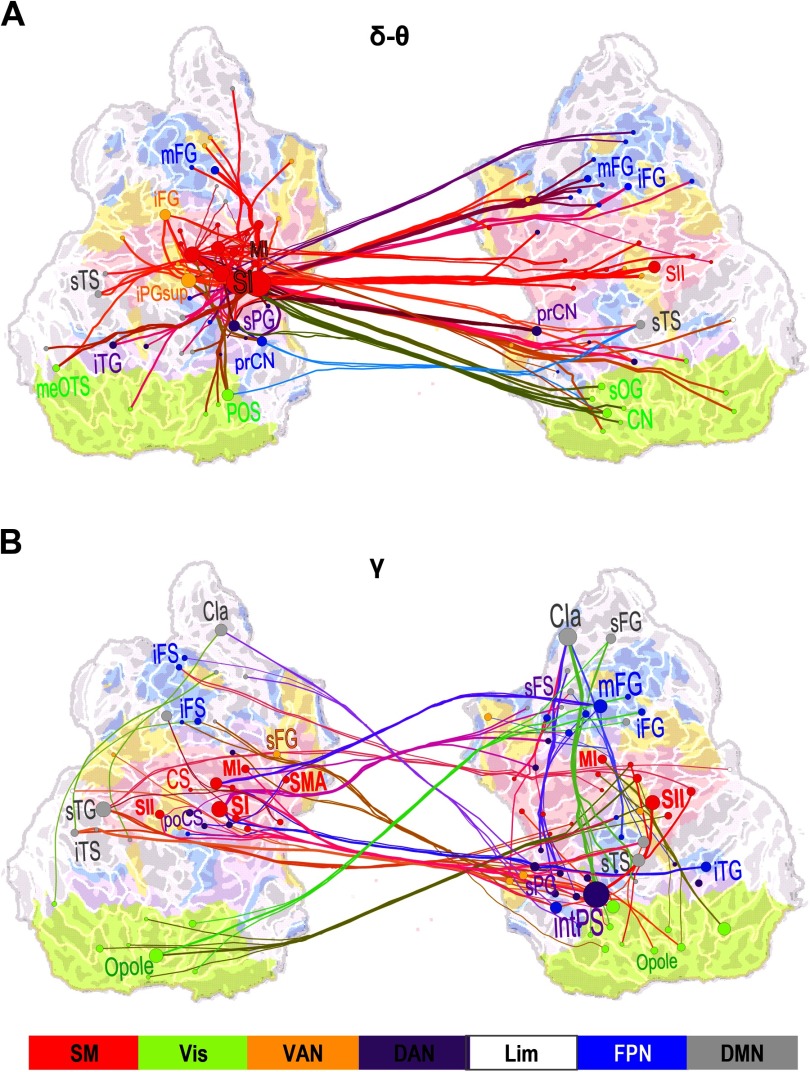
Large-scale synchronization connects somatosensory and attentional brain systems. (A) Graph of the significant differences in the strength of interareal phase synchrony as estimated with PLV between Hits and Misses in the delta/theta-frequency band (δ/θ, 3–7 Hz) and in the time window of 125–275 ms from stimulus onset (cf. Figure 2C). Lines connect the coupled parcels and line colors are determined by the parcel brain systems (see below). Delta/theta-band synchronization was centered on the contralateral (left hemispheric) sensorimotor (SM, red) system, and in particular, on the primary somatosensory cortex (SI) therein that was strongly and bilaterally coupled with frontal and parietal regions. (B) Graph of gamma-band synchronization (*γ*, 40–60 Hz) reveal significantly stronger connections for Hits than Misses over 225–375 ms from stimulus onset. Stronger gamma-band synchronization for Hits than Misses was observed within SM and between SM and the ipsilateral frontoparietal and dorsal (FP and DA, blue and purple) attention networks. Graphs are displayed on an inflated and flattened cortical surface with 300 (A) and 200 (B) of the most central edges based on parcel PageRank centralities selected for visualization. SI is primary and SII is secondary somatosensory area. MI and SMA are primary and supplementary motor areas, respectively. Parcel and corresponding node colors indicate the Yeo-atlas brain systems derived from BOLD intrinsic connectivity connectome. SM = somatomotor (SM), green = visual (Vis), yellow = ventral attention network (VAN), purple = dorsal attention network (DAN), white = limbic (Lim), gray = default mode network (DMN).

To identify the cortical areas connected by large-scale delta/theta- and gamma-band synchronization, we identified both the most central of the significant interareal connections and key cortical areas, that is, the network hubs, by using PageRank for those time-frequency windows where synchronization was significantly greater for Hits than for Misses. To obtain a coarse localizer for the functional subsystems of the cerebral cortex, we co-localized our cortical parcellations with the seven predominant systems defined by intrinsic fMRI BOLD signal correlations (Yeo et al., 2011). In the delta/theta band, the strongest connections for the difference between Hits and Misses were observed bilaterally in the sensorimotor (SM) system in the parcels corresponding to the contralateral primary sensory area (SI), secondary somatosensory areas (SII), and motor area (MI). Additionally, delta/theta-band synchronization connected SM with the bilateral middle frontal gyrus (mFG) in the dorsolateral prefrontal cortex (dlPFC) and with nodes in the visual cortex (Figure 5A). In the mid-gamma band, the most central connections were observed between contralateral SM, the most central hubs being SI, SII, and MI, and the ipsilateral mFG and bilateral inferior frontal gyrus (iFG) of the dPLFC as well as with the intPS of the PPC (Figure 5B). Functionally, mFG and iFG belong to the fronto-parietal network (FPN) whereas intPS belongs to the dorsal attention network (DAN). Gamma-band synchronization hence connected SM to FPN and DAN. In addition, mid-gamma-band synchronization connected mFG and intPS ipsilaterally.

### Large-Scale Synchrony Among Functional Subsystems

**Figure 6.**
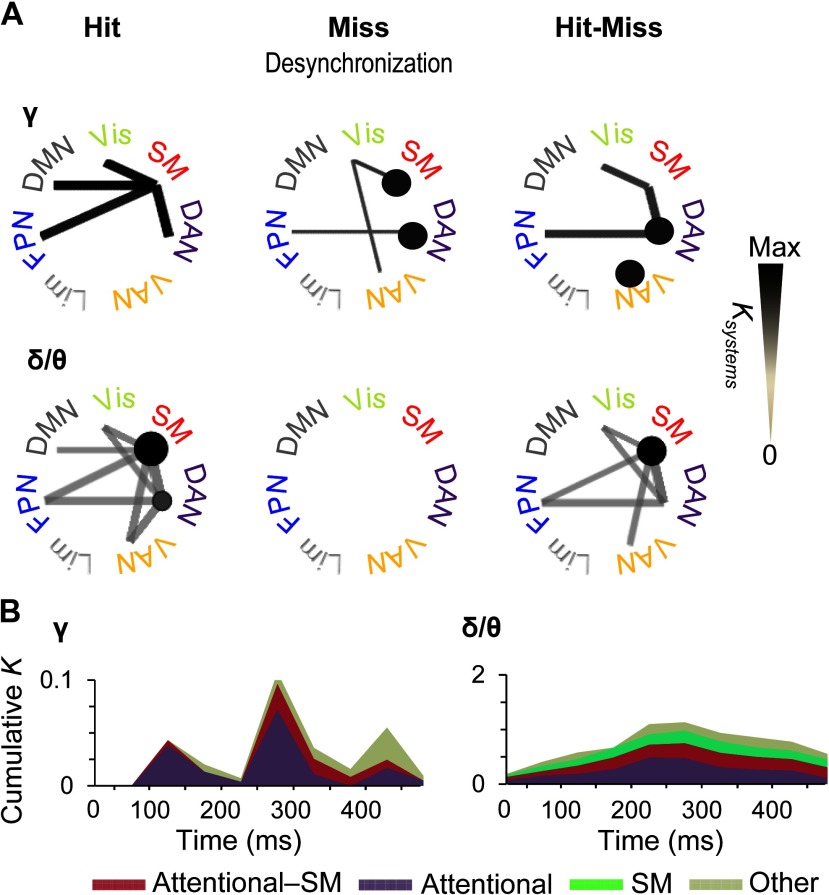
Time-varying subsystem connectivity within and between attentional systems and SM. (A) Connection densities of significant interareal gamma-band (40–60) Hz and delta/theta-band (3–7 Hz) synchronization among Yeo-atlas brain systems for Hits and Misses compared with baseline and for their difference. Only such system-system connections are shown that exhibit greater connection densities than expected by chance in shuffled graphs (*p* < 0.05, permutation statistics, see Methods). The color, line width, and radius of circles of the system-system connections indicates the connection density of significant couplings (*K*_systems_) within (circles) or between (lines) the functional systems in a time window of 225–375 ms. (B) Time-resolved cumulative connection densities (*K*) of gamma- and delta/theta-band synchronization estimated separately for within-attentional (DAN, FNP, and VAN), between SM and attentional, and all other functional subsystems. Synchronization in task-positive sensory and attentional systems predicted subsequent conscious perception in gamma band, whereas synchronization in the delta/theta band was observed in SM along with connections to and within-attentional systems throughout the time course of stimulus detection.

To complement the analyses of strongest connections, we obtained a systems-level view of all significant observations of interareal connectivity by identifying which functional brain systems were coupled by greater fractions of significant connections (*K*_*systems*_) than expected by chance (multiple-comparisons controlled graph permutation test, *p* < 0.05, see Methods). This analysis showed that for Hits, gamma-band synchronization connected the SM system with DAN and FPN as well as with the default mode network and visual system (Figure 6A). Interestingly, for Misses, the suppression of synchronization below baseline levels was found both within SM and DAN as well as among SM, ventral attention network (VAN), and the visual system, and between DAN and FPN. Hence, when the stimuli were not detected, attentional networks were uncoupled by suppression of gamma-band synchronization. In addition, the significant differences in synchronization between Hits and Misses were more prevalent between DAN and SM, within DAN and within VAN, and between DAN and FPN than expected for comparable random networks. Thus, these system-level results corroborate the findings of the most central connections and showed that gamma synchronization among somatosensory and attentional systems predicts stimulus detection in both the delta/theta- and gamma bands. Hit-related synchronization was found among SM and attentional systems for both frequency bands, the key difference being that in the delta/theta band, within-system synchronization was found in SM, whereas in the gamma band it was robust within DAN and VAN.

To illustrate the temporal evolution of the system-scale gamma synchronization and specifically to compare quantitatively the contributions of task-positive attention and sensory networks, we estimated the total connection densities of significant system-system couplings separately for within-attentional, attentional-sensorimotor, and all other systems (Figure 6B). Here, attentional systems included FPN, DAN, and VAN, whereas the “other” systems included the default mode, visual, and limbic systems. We found system-level gamma synchronization to emerge in three waves. In the first wave at around 100–150 ms from stimulus onset, connectivity was almost completely dominated by coupling within the attentional systems, with only a minor sensory-attentional fraction (Figure 6B, left). In the second wave at around 250–300 ms, coupling within-attentional systems remained predominant, but sensory-attentional coupling became much more salient. In the third wave, at around 450 ms from stimulus onset in which on average also the behavioral responses were given, connectivity in other systems was most prevalent. In contrast, delta synchronized emerged in one wave, which peaked between 200 and 300 ms after stimulus onset. Furthermore, it predominantly comprised synchronization within SM and attentional systems and between these two (Figure 6B, right).

### Single-Subject Statistics Corroborate The Robustness of Gamma-Band Synchronization in the Sensorimotor Decision Process

**Figure 7.**
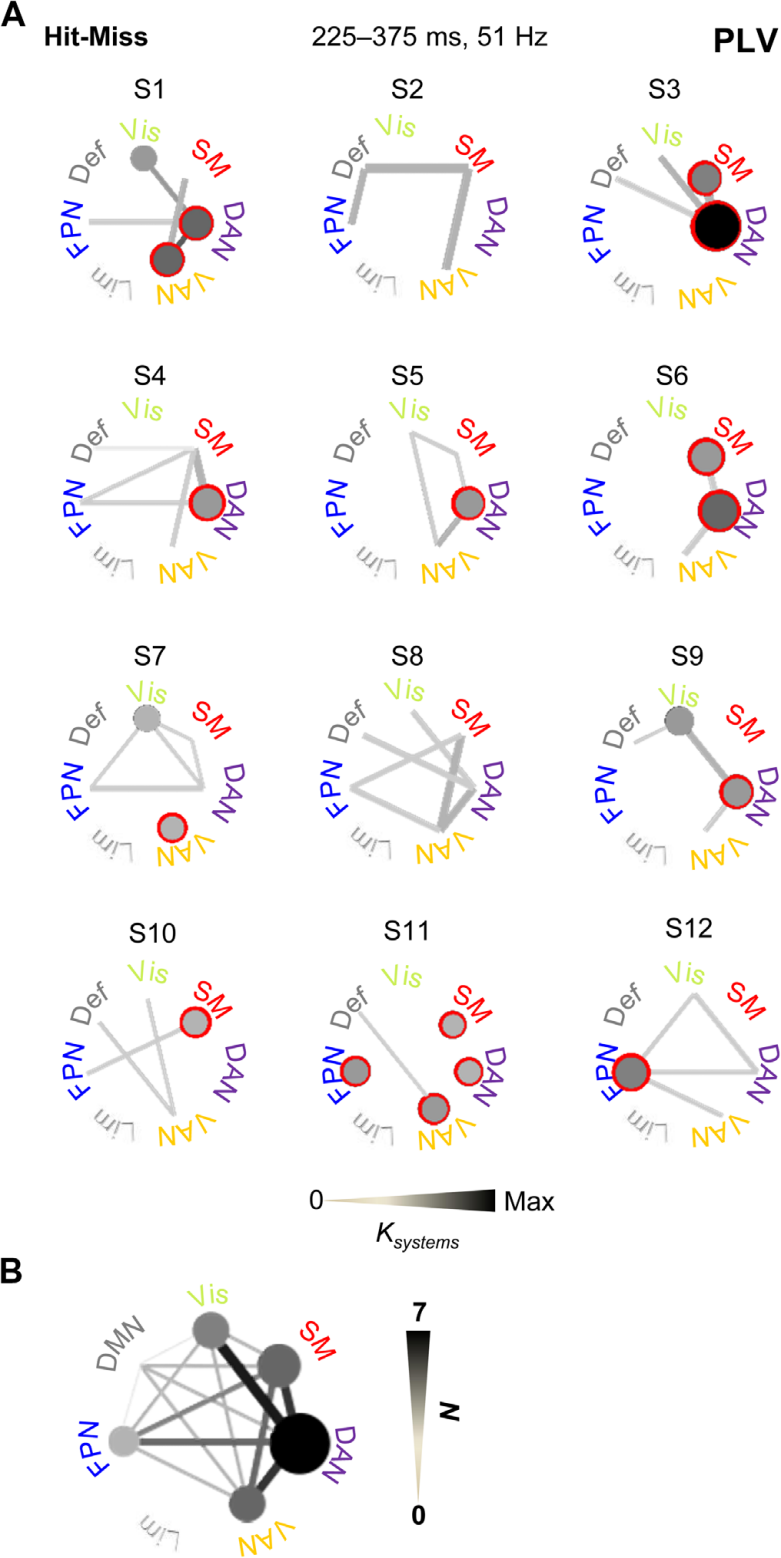
Synchrony within and between attentional and sensorimotor systems is frequently observed in individual subject’s statistical analyses. (A) All significant connections among functional subsystems separately for each subject. Significant connections are shown with gray tone that is scaled relative to the connection density between (lines) or within (circles) the subsystems. Red surroundings indicate the most common within-subsystem connections across the subjects. (B) All significant connections across all subjects in individual subject statistical analyses. Line color indicates the number of subjects (*N*) in which significant connections were were observed.

To corroborate the group-level analyses and to assess the robustness of gamma-band synchronization in single subjects, we investigated individual interareal synchrony with single-subject permutation statistics for the mid-gamma band (51 Hz) in the time window of 225–375 ms where synchronization was most extensive in the group data. We estimated subsystem-level connectivity as above separately for significant connections across subjects. This approach revealed that synchronization was most prominent within SM, FPN, DAN, VAN, and the visual system, and most repeatedly connected SM with DAN as well as DAN with VAN and the visual system (Figure 7A). Even with considerable interindividual variability, in 9 out of 12 participants the SM system was connected with an attentional system, and in all subjects synchronization was observed within and/or between these functional systems (Figure 7B). These findings thus confirmed that the correlation of large-scale gamma-band synchronization among SM, FPN, DAN, and VAN with perception is a robust phenomenon even in individual subjects.

## DISCUSSION

We aimed in this study to identify the large-scale networks of phase-locked cortical regions supporting the neuronal processing from perception to perceptual decisions and to actions. Building on accumulating evidence for the putative essential roles of large-scale neuronal interaction networks in cognitive integration (Baker et al., 2014; de Pasquale et al., 2015; Deco et al., 2015; Petersen & Sporns, 2015; Siegel et al., 2012) and of neuronal phase coupling in regulating the information transfer (Fries, 2015), we assessed the role of dynamic large-scale phase synchronization in the emergence of coordinated neuronal processing that accomplishes the transition from sensory perception to action generation.

This is the first study to show, using human source-reconstructed noninvasive MEG or EEG data, that dynamic interareal synchronization characterizes the neuronal activity cascade from perception of sensory stimuli, to perceptual decisions, and to actions. We revealed the spatiotemporal structure of this synchronization by using time-resolved synchrony analyses and localized the key cortical areas of synchronization networks. Importantly, we found that the strength of this synchronization was correlated with both detection performance (HR) and RTs, and hence is functionally significant.

We observed that two concurrent networks of transient synchronization, one in the delta/theta- and another in the gamma-frequency band, connected the sensory, fronto-parietal, and motor systems from perception to action. Delta/theta-band synchronization was pronounced within the SM system and between SM and attentional systems. In contrast, gamma-band synchronization was first observed within the fronto-parietal attention networks, and subsequently also between attention networks and the SM system, but it was not found to connect nodes within SM. These couplings connecting the key task-relevant cortical structures suggest that they could be functionally significant in the perception-action cycle. In line with their functional significance, the strength of delta-band and the timing of gamma-band synchronization were associated with the intertrial variability of the subsequent response times. These data thus demonstrate that the large-scale network interaction mechanisms achieving perception, perceptual decisions, and generation of actions may be achieved by phase coupling of neuronal oscillations in delta/theta and gamma bands.

These data now show that in addition to evoked responses and local modulations of oscillation amplitudes (Blankenburg et al., 2003; Hirvonen & Palva, 2016; Jones et al., 2007; King et al., 2016; Nierhaus et al., 2015; Taskin et al., 2008), somatosensory perception is also characterized by concurrent induced neuronal synchronization connecting the task-relevant cortical areas. In summary, these data provide evidence for the overarching hypothesis that neuronal synchronization may coordinate communication during cognitive operations (Fries, 2015) and that long-range synchronization may play a key role in the emergence of conscious sensory perception (Dehaene & Changeux, 2011; Engel & Singer, 2001; Tallon-Baudry, 2012) and sensorimotor decisions (Donner et al., 2009a; Engel & Fries, 2010).

### Long-Range Synchrony May Coordinate Processing from Perception to Action

The interest toward large-scale phase coupling as a putative mechanism for the emergent coordination of distributed processing into coherent cognitive operations stems from two established lines of evidence. First, phase coupling—synchronization—in a given neuronal assembly endows greater postsynaptic impact than internally uncoupled, incoherent assemblies (Konig et al., 1995; Singer, 1999). Second, interareally organized phase relationships or phase coupling may play a powerful role in opening or closing “channels” for communication among brain areas by facilitating communication during the high-excitability phases of neuronal oscillations (Fries, 2015). Cellular-level and animal model studies thus provided a strong mechanistic framework for the likely functional roles of interareal phase coupling. Nonetheless, because of significant methodological challenges, many of the resulting hypotheses have remained unaddressed at the level of large-scale cortical interaction networks and their relationship with human cognitive operations.

Prior fMRI-EEG studies have shown that activity in sensory and fronto-parietal brain areas is related to both conscious perception and generation of actions in response to somatosensory stimuli (Blankenburg et al., 2003; Nierhaus et al., 2015; Taskin et al., 2008), whereas EEG and MEG studies have shown that early evoked responses are stronger for detected somatosensory stimuli (Hirvonen & Palva, 2016; Jones et al., 2007; Nierhaus et al., 2015). Yet, the role of large-scale network synchronization in coordinating the perception and actions has remained poorly understood. A seminal study using intracranial EEG (iEEG) recordings from epileptic patients found that long-distance beta synchronization between frontal, parietal, and visual areas characterized visually presented words when these were consciously perceived but not if they remained undetected (Gaillard et al., 2009). Whether similar long-range synchronization would characterize also the perception of sensory stimuli in other modalities, in different experimental paradigms, and in MEG/EEG data obtained over the whole cortex has remained elusive.

Our central aim was to investigate whether large-scale networks of neuronal phase synchronization would be essential in the transformation of sensory processing to sensorimotor decision and actions. To this end, we used MEG recordings combined with several advanced data analysis approaches and graph theory (Bullmore & Sporns, 2009) to identify the spatiotemporal patterns of network synchronization from the perception of weak somatosensory stimuli presented at a threshold of detection to the action. We found that as hypothesized, dynamic large-scale synchronization characterized this process. Large-scale synchronization was observed concurrently in delta/theta and gamma bands showing that even the simplest perceptual process involves dynamic synchronization in multiple frequencies. Using a novel parcellation scheme (Korhonen et al., 2014), combined with the novel edge-bundling approach (Wang et al., 2018), we also identified the network structures of these networks. Delta/theta-band synchronization connected the contralateral SI with ipsilateral SI and SII bilaterally and thereby the brain regions underlying the processing of somatosensory information in monkeys (Haegens et al., 2014; Tauste Campo et al., 2015) and humans (Hegner et al., 2016; Hirvonen & Palva, 2016; Jones et al., 2007; Jones et al., 2009; Li Hegner et al., 2015; Papadelis et al., 2016). This connectivity pattern positions delta/theta-band synchronization to directly support the processing of somatosensory information. These nodes were also connected to the motor cortex (MI) as well as to the contralateral PFC supporting a close link and dynamic interactions between perceptual (Gutteling et al., 2011; Wohlschlager, 2000) and decision-making processes (Donner et al., 2009b; Gould et al., 2012; Kaplan et al., 2017; Tosoni et al., 2008) with the coordination of motor actions. Delta/theta-band synchronization could thus underlie the coordination of neuronal processing achieved collectively in motor and sensory cortices.

In contrast, gamma-band synchronization did not connect cortical areas within SM but coupled the contralateral SI and SII with ipsilateral sFS, mFG, and intPS. These areas are the key nodes in the fronto-parietal and DAN, FPN, and DAN, as indicated both by the whole-brain as well as by the subsystem analyses (Murakami & Okada, 2006; Power et al., 2011; Spadone et al., 2015), respectively. These FPN/DAN nodes were also mutually connected in both hemispheres, suggesting that gamma-band synchronization could here coordinate neuronal communication specifically within the attentional system and between the attentional and sensory systems. In summary, these data revealed many subnetworks among the key cortical areas that were correlated with conscious somatosensory perception, decisions, and actions in great anatomical and temporal precision. Unexpectedly, the data-driven all-to-all data analysis approach also revealed the visual system to be consistently connected with the sensorimotor system both in delta/theta and gamma bands. This may reflect the responsiveness of the visual cortex also to the somatosensory stimulation (Nordmark et al., 2012) and/or complementary representation of the perceived somatosensory stimuli in the visual cortex (Orlov et al., 2010), which is also plausible as the subjects had eyes closed during the task.

### Suppression of Gamma-Band Synchronization for Misses

Intriguingly, we found gamma-band synchronization to be suppressed below baseline levels for stimuli that remained undetected. These stimuli were hence subliminal in the terms that they modulated brain activity but in an inadequate manner to reach conscious perception (Dehaene et al., 2006; Dehaene & Changeux, 2011). Prior studies have reported that subliminal stimuli evoke neuronal activity in primary sensory cortices but not later in sensory processing hierarchy for visual stimuli (Dehaene et al., 2001; Del Cul et al., 2007; Grill-Spector et al., 2000). Our data showed that not only is the synchronization stronger for the consciously perceived and reported than for unperceived and unreported stimuli, but also that synchronization patterns between Hits and Misses are qualitatively and genuinely distinct. The suppression of gamma activity for Misses is, however, well in line with prior fMRI studies reporting negative BOLD fMRI signal responses in contralateral SI, SII, and SMA for subthreshold somatosensory stimuli (Blankenburg et al., 2003; Nierhaus et al., 2015; Taskin et al., 2008). Suppression of synchronization may reflect active uncoupling of task-relevant cortical areas to block conscious access from subliminal stimuli or it may reflect non-conscious stop-signals to interrupt motor responses (van Gaal et al., 2008).

### Temporal Pattern of Large-Scale Synchronization and Correlation with RT Supports Distinct Functions for Delta/Theta and Gamma-Band Synchronization in Perceptual Decisions and Actions

Finally, to reveal whether network synchronization would be stable or show dynamic changes as a function of time, we used time-resolved analysis of network synchronization. Interestingly, despite the stable profile of gamma-band synchronization in the time-frequency representations, this analysis showed that the gamma-band connectivity patterns changed as a function of time while those of delta-band synchronization pattern were more stable. During the early time windows, gamma-band synchronization connected nodes only within the attention networks, after which the gamma-band network comprised also the SM system, including motor cortex (M1). This temporal pattern suggests that in the present task, attentional systems drive sensory perception in line with prior studies, which suggest that perception is related to differences in the prestimulus network properties (Weisz et al., 2014; Frey et al., 2016) and predicted by infraslow fluctuations of electrical activity (Monto et al., 2008). Furthermore, fMRI BOLD signal fluctuations in attentional but not in sensory system are also correlated with perceptual performance in a threshold-stimulus detection task like ours (Sadaghiani et al., 2009). As PFC and PPC coordinate both perceptual decisions (Donner et al., 2009b; Goard et al., 2016; Hegner et al., 2016; Kaplan et al., 2017; Siegel et al., 2015; Tosoni et al., 2008) and conscious perception (Dehaene & Changeux, 2011; Gaillard et al., 2009; Hirvonen & Palva, 2016; Li Hegner et al., 2015; Salti et al., 2015), we speculate that gamma-band synchronization during the later time windows, however, could underlie conscious perceptual decisions of the weak somatosensory stimuli. The role of gamma-band synchronization in coordinating perceptual decisions was also supported by the distinct temporal profiles of synchronization for fast and slow actions. Interestingly, fast and slow responses were associated with distinct gamma-band patterns, which illustrates the presence of large intertrial variability in the functionally significant gamma-band synchronization patterns. More specifically, the neuroanatomical and temporal patterns of gamma-band synchronization could plausibly reflect a sequence of network reconfigurations from conscious sensory decisions to the coordination of motor actions.

In contrast, the temporal profile of delta/theta-band synchronization was more stable. This may be caused by more stable network configuration of synchronization or the poorer temporal resolution of wavelets compared with the gamma band. Synchronization was confined to SM system across time. This together with an increase in the strength of synchronization but not a change in temporal profile in response to faster RTs suggest that delta-band synchronization is related to evidence accumulation of sensory information and coordination of motor actions but not the achieving conscious sensory perception per se. Overall, in line with prior studies of local neuronal processing in humans (Donner et al., 2009b; Gould et al., 2012; Kaplan et al., 2017; Medendorp et al., 2011; Tosoni et al., 2008) our data suggest that the sensorimotor decision process and the associated actions involve overlapping neuronal circuits also at the large-scale network level.

### Conclusions

In summary, these findings constitute evidence for the hypothesis that dynamic large-scale network synchronization plays an essential role in the coordination of neuronal processing to achieve conscious somatosensory perception, perceptual decisions, and initiation of a motor response.

## METHODS

An overview of the workflow is given in Figure 1. All analyses, if not stated otherwise, were analyzed with LabVIEW software (National Instruments), available on request.

### Subjects and Recordings

MEG was recorded from 12 healthy right-handed subjects (27.5 ± 4.5 years of age, mean ± standard deviation; 7 women) with a 306 channel MEG (Elekta Neuromag, Helsinki, Finland) at 600-Hz sampling rate as described earlier in (Hirvonen & Palva, 2016). Electromyogram (EMG) was recorded to detect the thumb movement responses and T1-weighted anatomical MRI scans were obtained for source localization. The study was approved by the Coordinating Ethical Committee of the Helsinki University Hospital, and the subjects gave a written, informed consent prior to their participation in the experiment.

### Task

We used a continuous stimulus detection task in which 0.2-ms electrical stimuli were given with an intensity (mean 4.3 ± 0.6 mA) at the threshold of detection (Hirvonen & Palva, 2016; Monto et al., 2008; Palva et al., 2005). The intensity was individually calibrated before the recordings to a level that yielded a ∼50% detection rate and then maintained constant during the recordings. The stimuli were delivered at uniformly random 1.5- to 4.5-s intervals to the right index finger in two separate approximately 30-min blocks. The subject’s task was to report with a right thumb twitch whenever he or she perceived the stimulus. On average, 1,073 ± 207 (mean ± SD, *n* = 12) trials were acquired per subject. No trials were rejected.

### Behavioral Performance

HR was defined to be the proportion of detected stimuli of all stimuli. The stimuli were classified as consciously perceived, or “Hits,” if the subject performed the right thumb twitch response within 0.1–1.5 s after the stimulus onset and “Misses” if stimulus was not associated with response (Figure 2A). The responses were identified from filtered (low- and high-pass finite impulse-response filter with pass band 30–190 Hz) and absolute valued EMG recordings using 10 baseline standard deviations as the threshold for a response and the first crossing of this threshold as the RT (Hirvonen & Palva, 2016; Monto et al., 2008; Palva et al., 2005). Stimuli to which no response was observed were categorized as unperceived, or “Misses.” To estimate the false alarm (FA) rate, we estimated the number of responses between −1.5 s to 0 s before stimulus onset, during which there should not have been any responses to somatosensory stimuli. For this estimation, only the trials with a distance at least 3 s to previous and next trial were selected. FA estimate was then computed as the proportion of these responses from all the stimulus events (Figure 2B). Delayed responses and FA were not analyzed because of the exceedingly small number of trials in these categories.

### MEG Data Preprocessing, Filtering, and Source Analysis

The signal space separation method (tSSS) was used to remove extracranial noise from the raw MEG recordings, and independent component analysis was used to identify and exclude components associated with eye movements and cardiac artifacts (Figure 1, a). In short, preprocessed MEG sensor time series were filtered using Morlet wavelets into 38 frequency bands covering 3–120 Hz with equal distances between neighboring frequencies on the log scale by using time-frequency compromise parameter *m* = 5 (Figure 1, b). We used FreeSurfer software (http://surfer.nmr.mgh.harvard.edu/) and minimum-norm estimate (MNE) toolkit for volumetric segmentation of MRI images, and reconstruction of anatomical surfaces and cortical parcellation (Destrieux et al., 2010; Fisch et al., 2002) (Figure 1, c). The MNE toolkit (http://www.nmr.mgh.harvard.edu/martinos/userInfo/data/sofMNE.php) was used to create three-layer boundary element models, cortically constrained source models, MEG-MRI co-localization and for preparation of the forward model and MNE inverse operators (Hamalainen & Ilmoniemi, 1994) (Figure 1, d). The source models had dipole orientations fixed to the pial surface normals and a 7-mm source-to-source separation throughout the cortex, which yielded models containing 6,000–8,000 source vertices.

We used the MNE inverse operators in the form of dynamic statistical parametric map (dSPM) operators (Dale et al., 2000) so that the noise-covariance matrices (NCM) were obtained from the baseline data 0.75–0.2 s prior to stimulus onsets and by using 0.05 as the regularization constant. The NCMs and hence the inverse operators were prepared separately for each Morlet-wavelet filter frequency by using lambda as 0.05. We used an atlas-based analysis strategy (Hillebrand et al., 2012; Palva et al., 2010), in which the preprocessed and Morlet-filtered MEG sensor time series were inverse modeled sample-by-sample into source time series (Figure 1, d) that were then collapsed into time series of 400 cortical parcels by using individually optimized collapse operators (Figure 1, e) (Korhonen et al., 2014). In these collapse operators, the source vertices were weighted with parcel-signal reconstruction accuracy, and only the set of vertices that yielded best reconstruction accuracy were used (Korhonen et al., 2014). For creating these weighted collapse operators, and more precisely, to assess parcel-signal reconstruction accuracy, another set of NCM and inverse operators were computed. To obtain NCM only for this “fidelity optimization” inverse-forward modeling, we applied 0.1–45 Hz pass-band filtering to MEG time series by using low- and high-pass finite impulse-response filters. The 400-parcel parcellation was obtained from a precursor “Destrieux” atlas of 148 parcels (Destrieux et al., 2010) by iteratively splitting the largest parcels along their most elongated axis and using the same parcel-wise splits for all subjects. Using neuroanatomical labeling as the anatomical “coordinate system” eliminates the need for intersubject morphing in group-level analyses, which would have compromised individual anatomical accuracy.

### Forward and Inverse-Modeled Trial-Shuffled Surrogates for Evoked Component Estimation

To account for the artificial synchronization attributable to evoked responses and/or phase locking of ongoing activity to the stimuli, we created trial-shuffled surrogate data (Figure 1, f). To reconstruct the effects of signal mixing at MEG acquisition and inverse modeling, we applied a forward-inverse modeling–based surrogate construction approach that reconstructs the evoked components and their spatial spread caused by the signal mixing inherent to MEG/EEG and preserves local source topography changes, amplitude dynamics, and changes in autocorrelation structures that may constitute significant confounders (Palva & Palva, 2012). This approach hence provides a good surrogate for selectively identifying the true induced interareal interactions for which the conventional trial shuffling (Lachaux et al., 1999) is insufficient in the presence of signal mixing.

We used the source-modeled single-trial data in the 400-parcel parcellation as a new parcel time series so that first in forward modeling the activity time series of each source vertex of a given parcel for each trial was simulated with the parcel time series of a random trial. In each new “trial” of the surrogate data, the cortical parcels were simulated with random-trial time series of the original data. We then source reconstructed these sensor-level surrogate data with procedures identical to those used for real data. This procedure thus abolishes true non-stimulus-locked phase correlations between parcels but reconstructs both the evoked and stimulus-phase-locked components as well as the spread of the signals caused by MEG data acquisition and inverse modeling. Phase correlation analyses (Figure 1, g) were then performed with these surrogate source data identically to those of real data for 10 independent realizations of the surrogate data. The means of surrogate data were compared against the corresponding real data in Fig. 3 (Figure 1, f).

### Analysis of Phase Synchrony

All-to-all phase coupling–based functional connectivity, that is, phase synchrony, between all parcel pairs, was estimated in reconstructed source space for all 38 narrow-band time series. We first estimated the complex-valued phase-locking value (cPLV) between all parcel pairs. The cPLV is defined as

$$cPLV=\frac{1}{N}\sum_{n=1}^{N}\left[e^{i\left(\theta_{X}(n)-\theta_{Y}(n)\right)}\right]$$ (1)

where *N* denotes sample number, *θ*_*X*_ and *θ*_*Y*_ are phases of narrow-band time series of *X* and *Y*, and *I* denotes the imaginary unit (Lachaux et al., 1999; Palva et al., 2005). We assess here phase synchrony both by using the PLV, PLV = |cPLV|, and the imaginary part of cPLV (iPLV), iPLV = |*im*(*cPLV*)|, where *im* indicates the imaginary value operator. PLV, a commonly used measure for phase correlations, is essentially equally sensitive to phase coupling at all phase lags and thus is sensitive to artificial zero-phase-lag correlations caused by MEG signal mixing and spatial leakage in inverse modeling. iPLV, on the other hand, is insensitive to near-zero and near-pi phase lags and thus does not yield false positives attributable exclusively to signal mixing (Brookes et al., 2014) (Figure 1, g).

To compensate for the fact that both PLV and iPLV are biased by the number of samples, the numbers of Hit and Miss trials were balanced within subjects before the PLV/iPLV analyses by keeping only those events of the larger conditions, which are closest to the onset latencies of the smallest condition. For each frequency, the cPLV values were obtained across samples in 100-ms time windows and across trials. Using 50% overlap, we obtained 26 time windows from −725 ms to 525 ms around the stimulus.

### Group Statistical Analyses

Group statistical contrasts between conditions or between prestimulus baseline and poststimulus periods were performed separately for each frequency, time window, and parcel pair. Before group statistics, mean baseline values from 125 to 225 ms prior to stimulus onset were subtracted. The significance of the difference between stimulus processing and baseline or between Hits and Misses was estimated with the Wilcoxon signed-rank test (*p* < 0.05). To reduce false discoveries arising from multiple comparisons, we pooled significant observations for each contrast across all cortical parcels and time windows but separately for each frequency band. Then we discarded as many least significant observations across parcels and across time windows as were predicted by the alpha level (α) and the total number of comparisons (*N*_*c*_), α × *N*_*c*_ (Figure 1, h). This false discovery reduction was done separately for each frequency band.

### Removal of Parcels and Connections with Low Reliability

The poor source reconstruction accuracy of some cortical parcels limits the overall quality of all-to-all interaction analyses. In this study, we utilized a simulation-based method to assess the reliability of local source time-series reconstruction and interareal interaction estimates (Korhonen et al., 2014). We used the subject cohort’s source models, that is, forward and inverse operators that were derived from real MEG recordings, simulated independent parcel time series as the ground-truth data, and then simulated a virtual MEG recording and source reconstruction by forward and inverse modeling the ground-truth time series. Thus, assessing the correlations between simulated and reconstructed data yields quantitative estimates of the reconstruction accuracy and signal mixing between parcels. We used parcel *fidelity* (*f*_*u*_) to quantify the source reconstruction accuracy of a parcel *u*; it is defined as follows:

$$f_{u}=\left|re(cPLV(x_{u},{\hat{x}}_{u}))\right|$$ (2)

where *x*_*u*_ is the simulated original time series of parcel *u*, and ${\hat{x}}_{u}$ is the source-reconstructed time series of *x*_*u*_.

We used parcel-to-parcel *infidelity* (*i*_*uv*_) to quantify the amount of mixing from parcels *u* to another parcel *v*, and it is defined as follows:

$$i_{uv}=\left|re(cPLV(x_{u},{\hat{x}}_{v}))\right|$$ (3)

where *x*_*u*_ is the original time series of parcel *u*, and ${\hat{x}}_{v}$ is the forward- and inverse-modeled time series of another parcel *v*. Thereby *i*_*uv*_ quantifies the amount of signal from *u* that is observed in the reconstructed signal of another parcel *v*, thus describing degree of signal leak from parcel *u* to parcel *v*. To decrease the probability of reporting artificial and spurious synchronization due to poor source reconstruction accuracy, we first removed parcels with fidelity lower than 0.11. These parcels were located mostly in deep and/or inferior parts of the cortex and are known to generate weak signals in M/EEG (Supporting Information Figure 1, Hirvonen, Monto, Wang, Palva, & Palva, 2018). Next, we excluded cortical parcels close to the eyes because they are known to include oculomotor artifacts in MEG. These parcels are mostly located afar (e.g., >5 cm) from the sensors such as the orbital frontal, anterior, and inferior temporal and medial structures. In total, 20% of such parcels were excluded from analyses. In addition, we also excluded parcel-parcel interactions that connected any parcels with low *fidelity*, that is, interactions that would be very unlikely to be observable with MEG. In analyzed data, these couplings would be thus much more likely to mirror signal-mixed interactions from other sources and thus yield false positives. Taken together, 38% of interactions were eliminated as being likely to be contaminated by poor reconstruction accuracy and linear mixing. (Figure 1, i).

### Visualization

To summarize our results, we used connection density, *K*, as a function of frequency bands to indicate the fraction of statistically significant edges in a given experimental-condition contrast (Figure 3). These data were used to identify frequency bands of interest (Figure 1, j). The graphs (Figure 5) corresponding to these data were then visualized so that most central connections were identified by the mean PageRank centralities of the connected nodes (Rubinov & Sporns, 2010). For graph visualization, we first evaluated the centrality of each edge *e*_*i*_(*u*, *v*) in the graph by summing PageRank centrality of the source and target nodes:

$$C{}_{ei}=sum(PR_{u}+PR_{v})$$ (4)

where PR denotes the PageRank centrality. The PageRank is a measure of the importance of a node, and therefore the *C*_*ei*_ measure weights more to edges that connect important nodes in a graph. Subsequently, we applied a novel hyperedge bundling approach to group edges into bundles by their adjacency in signal mixing (Wang et al., 2018), which was derived from the parcel-to-parcel infidelity function *i*_*uv*_ described earlier (Figure 1, k). Briefly, this approach bundles together edges that have large-signal mixing and hence which are spurious and artificially induced by the true neuronal connection. Thus, the hyperedge represents the under lying true interaction and its spurious reflections caused by signal spatial leakage. We excluded bundles of less than four edges, because true interareal synchrony is likely to be reflected in large numbers of signal spread caused by spurious connections around each true connection. To improve the neuroanatomical resolution of the bundles, we discarded edges with low centrality (<50%) within each bundle. The starting number of edges was selected so that the final edge number became 300 and 200 for graphs in Figure 5 so that the largest number of edges was selected for graphs with largest *K* and smallest for smallest *K*.

To estimate interactions between the seven functional subsystems (Yeo et al., 2011), we morphed the original 400 × 400 adjacency matrices into 7 × 7 subsystem interaction matrices (Figures 6A and 7), and evaluated *K* of connections among each subsystem (Figure 1, l). To test whether these *K* were greater than expected by chance for a random graph, we computed 5,000 randomizations of the same 400 × 400 matrices, keeping the numbers of significant edges constant. *K* values of the original subsystems interaction matrix were reported as significant if they exceeded the 95th percentile of the *K* values in the randomized graphs.

### Individual Subject Statistics

To estimate statistical significance of synchronization of the center frequency that was identified in the group-level analysis in single subjects, we used nonparametric permutation statistics (Figure 1, m). We computed the difference in mean PLV between Hits and Misses, δ*I*, within each time window and for each pair of parcels. To obtain the permutation-based statistics, we randomly redistributed the trials into Hit_perm_ and Miss_perm_ and computed δ*I*_perm_, the mean PLV between the permuted conditions. The random permutation and PLV recomputation was repeated for *N*_perm_ = 5,000 times. To obtain the subject-wise permutation test statistic, *p*_perm_, we counted the portion of permutations that resulted in higher absolute difference in PLV than the unpermuted conditions:

$$p_{\text{perm}}=\#(|\delta I_{\text{perm}}|>|\delta I|)/N_{\text{perm}},$$ (5)

where #() denotes the counting operation. The *p*_perm_ statistic was then corrected for predicted false discovery rate as explained above using α = 0.05 and *FDR* = 0.05.

## ACKNOWLEDGMENTS

We thank Dr. Alexander Zhigalov and MSc Santeri Rouhinen for contributing analysis and visualization tools.

## AUTHOR CONTRIBUTIONS

Jonni Hirvonen: Data analysis; Visualization; Funding aquisition; Manuscript writing. Sheng Wang: Software design. Simo Monto: Data collection; Software design; Data analysis; Visualization; Manuscript writing. Satu Palva: Experimental design; Data collections; Funding aquisition; Manuscript writing. Matias Palva: Experimental design: Software design; Funding aquisition; Manuscript writing.

## FUNDING INFORMATION

This work was supported by the University of Helsinki Research Grants, Academy of Finland (SA 267030, SA 266402 and SA 273807 to SP and SA 253130 and 256472 MP), Research Foundation of the University of Helsinki to SP and JH, and Sigrid Juselius Foundation to SP and MP for supporting this study.

## TECHNICAL TERMS

Magnetoencephalography (MEG): Measures magnetic fields produced by coherent current flows in neuronal populations.
Source reconstruction: Inverse modeling of MEG sensor data to estimate the underlying cortical source current distribution and time series.
Phase synchronization: Indicates a consistent phase relationship between oscillations, here between different brain areas.
Connection density: A proportion of connections in a graph out of all possible connections; here connections were defined by their statistical significance.
Parcellation: Division of the cortical surface into parcels that here correspond to neuroanatomically named brain areas.
Phase-locking value (PLV): The absolute value of complex PLV, quantifies the strength of phase synchronization.
Imaginary part of complex PLV (iPLV): A phase synchronization measure that is sensitive only to nonzero and non-pi phase lag couplings and thereby excluded the direct effects of linear mixing.
Evoked/stimulus-locked activity: A cortical response with a consistent latency after stimuli and thus observable in trial-averaged response.
Subsystem: A set of brain areas defined by intrinsic connectivity and typically corresponding to areas with associated functional roles.
