# Supplementary material for: Dynamic large-scale network synchronization from perception to action
Source: Netw Neurosci. 2018 Oct 1;2(4):442–63. doi: 10.1162/netn_a_00039 (PMC6175692; doi:10.1162/netn_a_00039)
Supplement: Supplementary file 1 [file netn-02-442-s001.pdf]

Hirvonen, J. Monto, S., Wang, S. H., Palva, J.M., & Palva, S. (2018). Supporting Information for "Dynamic large-scale network synchronization from perception to action." *Network Neuroscience,* 2(4), 442-463. https://doi.org/10.1162/netn_a_00039

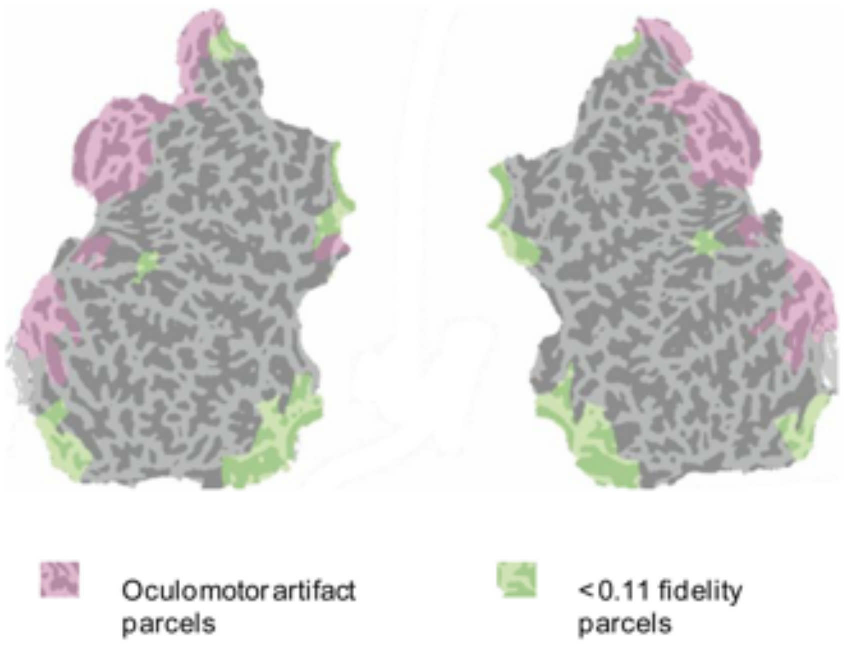

Supplementary Figure 1. 81 cortical parcels out of total 400 were excluded from synchronization visualizations owing to low fidelity (below the threshold of 0.11, neon) or staining by oculomotor artifacts (magenta).

Hirvonen, J. Monto, S., Wang, S. H., Palva, J.M., & Palva, S. (2018). Supporting Information for "Dynamic large-scale network synchronization from perception to action." *Network Neuroscience*. Advance publication. https://doi.org/10.1162/netn_a_00039

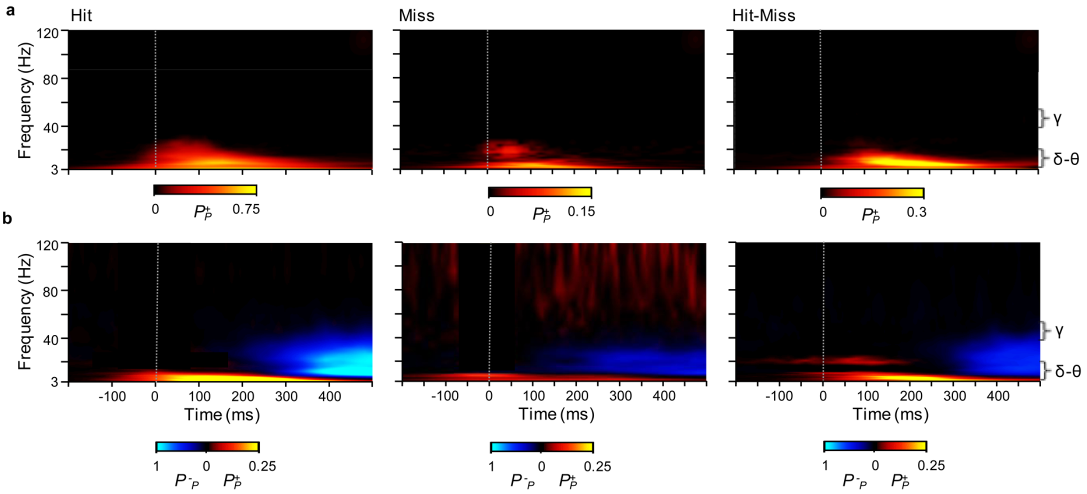

Supplementary Figure 2. A) Time-frequency representation (TFR) of narrow-band phase-locking of ongoing oscillations to stimulus onset separately for Hits, Misses and for their difference. The color scale indicates the fraction of parcels in which SL was stronger than in the pre-stimulus BL for Hits, Misses, and for their difference as a function of time and frequency (Wilcoxon signed rank test, $p < 0.01$, FDR corrected). B) TFR of the narrow-band induced oscillation amplitude modulations to Hits, Misses and for their difference.
